# Supplementary material for: The anatomy of past abrupt warmings recorded in Greenland ice
Source: Nat Commun. 2021 Apr 8;12:2106. doi: 10.1038/s41467-021-22241-w (PMC8032679; doi:10.1038/s41467-021-22241-w)
Supplement: Supplementary file 1 — Supplementary Information [file 41467_2021_22241_MOESM1_ESM.pdf]

The anatomy of past abrupt warmings recorded in Greenland ice

E. Capron[1,2#], S. O. Rasmussen[1], T. J. Popp[1], T. Erhardt[3], H. Fischer[3], A. Landais[4], J. B. Pedro[1,5,6], G. Vettoretti[1], A. Grinsted[1], V. Gkinis[1], B. Vaughn[7], A. Svensson[1], B. M. Vinther[1], J. W. C. White[7]

[1] Physics of Ice, Climate and Earth, Niels Bohr Institute, University of Copenhagen, Tagensvej 16, 2200 Copenhagen, Denmark.

[2] Université Grenoble Alpes, CNRS, IRD, IGE, 38000 Grenoble, France.

[3] Climate and Environmental Physics, Physics Institute & Oeschger Center for Climate Change Research, University of Bern, Sidlerstrasse 5, 3012 Bern, Switzerland.

[4] Laboratoire des Sciences du Climat et de l'Environnement, LSCE/IPSL, CEA-CNRS-UVSQ, Université Paris-Saclay, Gif-sur-Yvette, France.

[5] Australian Antarctic Division, Channel Highway, Kingston, Tasmania.

[6] Australian Antarctic Program Partnership, Institute for Marine and Antarctic Studies, University of Tasmania, Hobart, Tasmania.

[7] Institute of Arctic and Alpine Research, University of Colorado, Boulder, Colorado 80309-0450, USA.

[#]*Corresponding author: E. Capron, email: emilie.capron@univ-grenoble-alpes.fr*

Supplementary Information

Supplementary Table 1. Search intervals used for the ramp-fitting analysis on each abrupt transition covered by ice core data and shown in this study.

**Transition toward:**	**Search intervals for ramp-fitting analysis (yr b2k)**	
Holocene	11453	11953
GI-1e	14442	14942
GI-2.2	23240	23590
GI-3	27630	28030
GI-4	28650	29150
GI-5.2	32250	32750
GI-7c	35230	35730
GI-8c	37970	38470
GI-10	41210	41710
GI-11	43090	43590
GI-12c	46610	47110
GI-14e	54070	54470
GI-15.1	54920	55320
GI-15.2	55550	56050
GI-16.2	58190	58480
GI-17.1c	58880	59280
GI-17.2	59340	59640
GI-18	63900	64350
GI-19.1	69470	69870
GI-19.2	72090	72590
GI-20c	76190	76690
GI-21.1e	84560	84960
GI-22g	89840	90240
GI-23.1	103840	104240
GI-25a	110700	111190

Supplementary Table 2. Results of the test regarding the significance of the correlation between NGRIP and NEEM transition durations considering seven different groups of data: $\delta^{18}O$ transitions only, d-excess only, [Ca^{2+}] transitions only, [Na^{+}] transitions only, both $\delta^{18}O$ and d-excess transitions (referred to as Water isotope transitions), both [Ca^{2+}] and [Na^{+}] transitions (referred to as Impurity transitions) and finally considering the transitions in all tracers together.

**Correlation between**	**N**	**Frequency $\|s\|>s_0$**
$\delta^{18}O$ transitions only	24	0.0311
d-excess transitions only	13	0.0623
Ca^{2+} transitions only	22	0.178
Na^{+} transitions only	18	0.0229
Water isotope transitions only	37	0.00055
Impurity transitions only	40	0.008
All transitions	77	0.00003

Supplementary Table 3. Six modeled abrupt events from three simulations run under different prescribed atmospheric CO_2 concentrations with the low-resolution version of CCSM4. Search intervals used for the ramp-fitting analysis on the time series of simulated climate parameters are also indicated.

**Transition toward:**	**Prescribed CO_2 concentrations**	**Search intervals for ramp-fitting analysis (yr)**	
Modeled event 1	185 ppm	3000	3500
Modeled event 2	185 ppm	4750	5250
Modeled event 3	200 ppm	2750	3250
Modeled event 4	200 ppm	5300	5800
Modeled event 5	210 ppm	3100	3600
Modeled event 6	210 ppm	5350	5850

Supplementary Figure 1. High-resolution NGRIP and NEEM ice core records over 600-yr-long windows covering the studied abrupt transitions: $\delta^{18}O$ from NGRIP (dark blue) and NEEM (light blue) (ref. 1, this study), d-excess from NGRIP (red) and NEEM (orange) (this study), annually-resampled log (Ca^{2+}) from NEEM (dark green) and NGRIP (khaki green) (ref. 2; this study) and NGRIP annual layer thickness (grey, ref. 3). Onset points, end points (symbols) and ramps (oblique lines) together with associated uncertainty intervals (horizontal shaded lines) found by the ramp-fitting analyses (this study) are indicated. Vertical dashed lines indicate the search interval for the ramp-fitting tool.

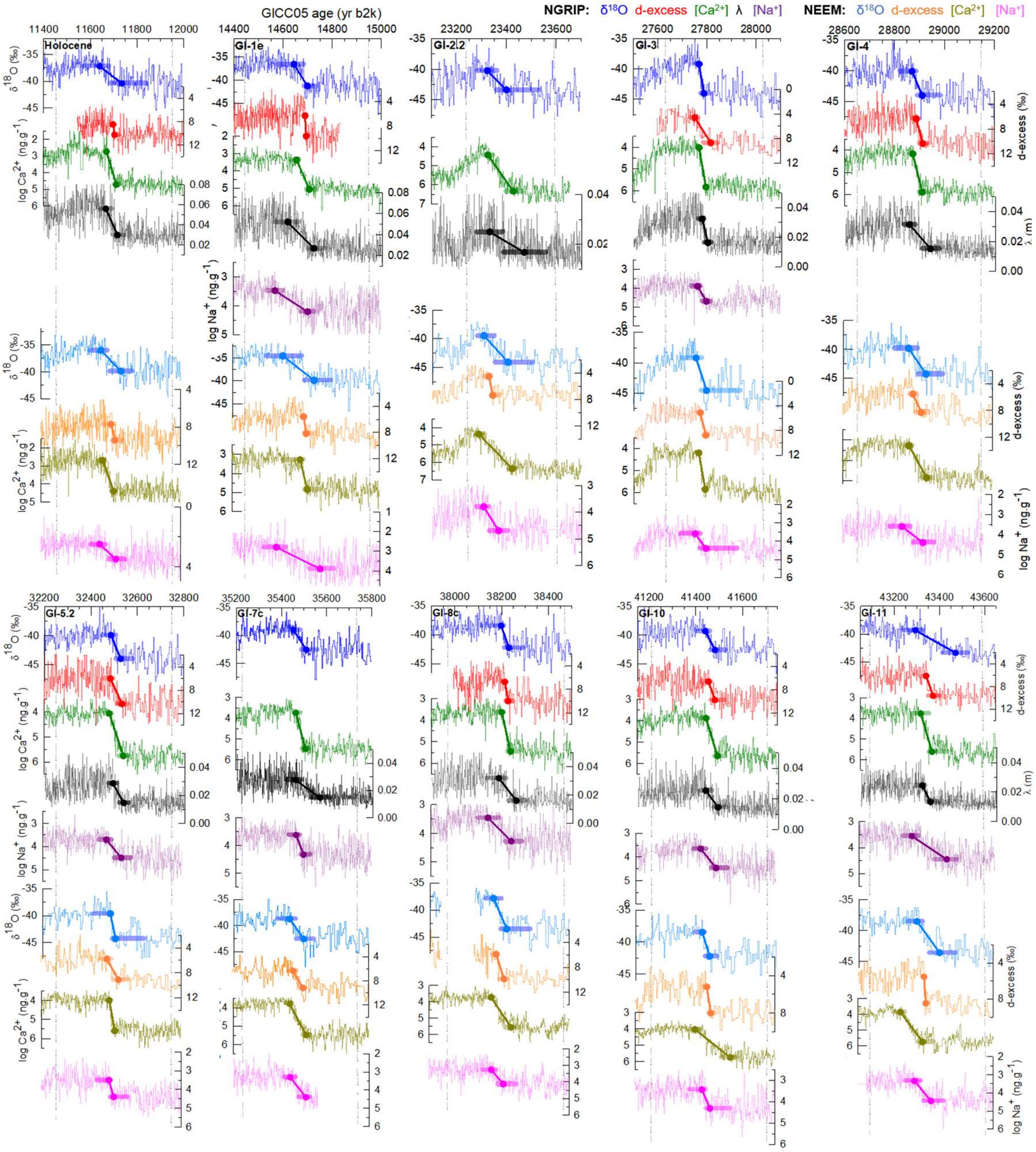

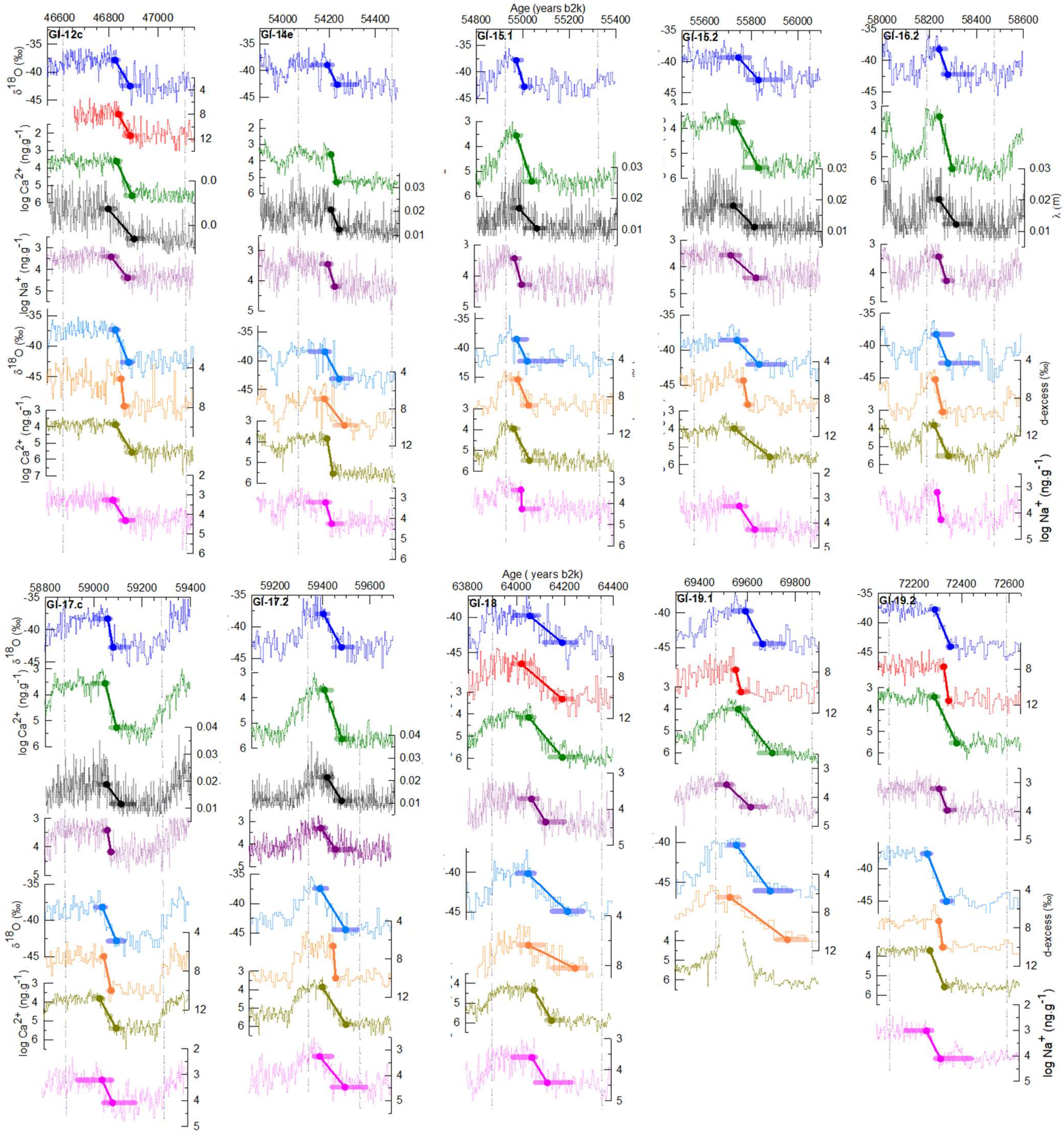

Age (years b2k)
GI-12c
GI-14e
GI-15.1
GI-15.2
GI-16.2
GI-17.c
GI-17.2
GI-18
GI-19.1
GI-19.2
Age (years b2k)
δ18O (‰)
log Ca2+ (ng.g-1)
log Na+ (ng.g-1)
λ (m)
d-excess (‰)

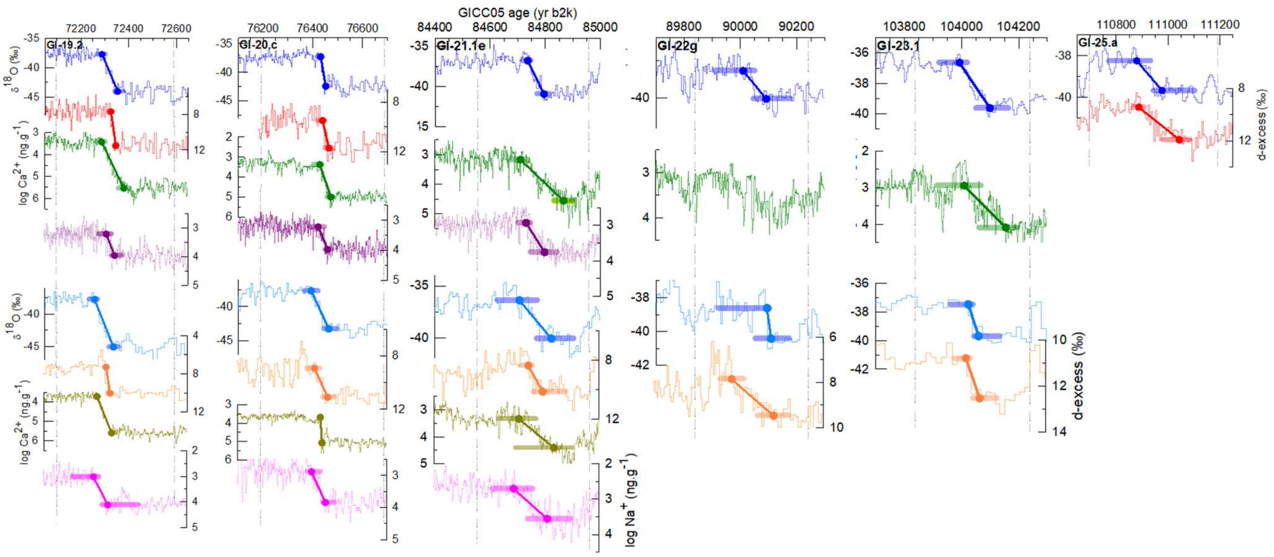

GICC05 age (yr b2k)
GI-19.2
GI-20.c
GI-21.1e
GI-22g
GI-23.1
GI-25.a
δ18O (‰)
log Ca2+ (ng.g-1)
log Na+ (ng.g-1)
d-excess (‰)

Supplementary Figure 2. Test of ramp fitting in the presence of autocorrelated noise. 20 different artificial noisy ramps were analysed with our ramp fitting method and the resulting transition durations for each iteration (open blue circle) are displayed together with the marginal posterior 5-95% credible intervals (vertical blue bars). The true duration of 50 years (red dashed line) is within the 5-95% credible intervals in 19 of the 20 cases.

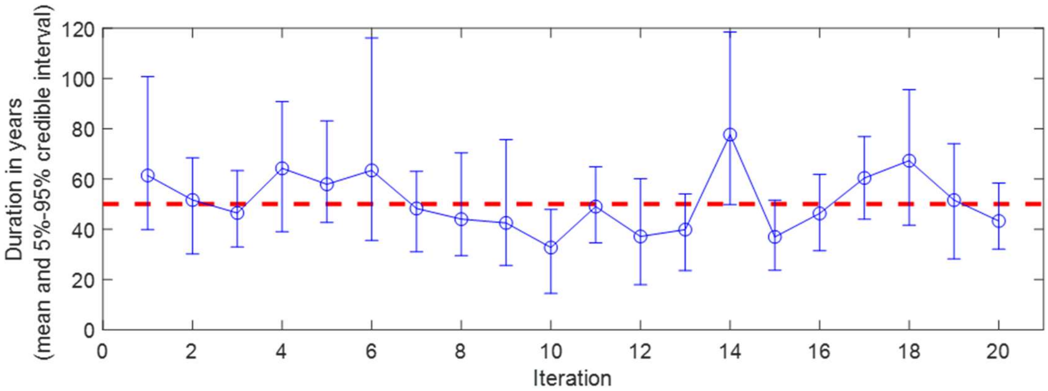

Supplementary Figure 3. Sensitivity tests on the NGRIP $\delta^{18}O$ transitions into GI-6 (left panels) and GI-12c (right panels). The bold blue ramps are defined by the ramp-fitting model run over three search windows of different widths: (a, d) ±100 yr, (b, e) ±200 yr and (c, f) ±250 yr. The durations of the resulting transitions are indicated in blue and the used search window is indicated by the horizontal black arrows and the white boxes.

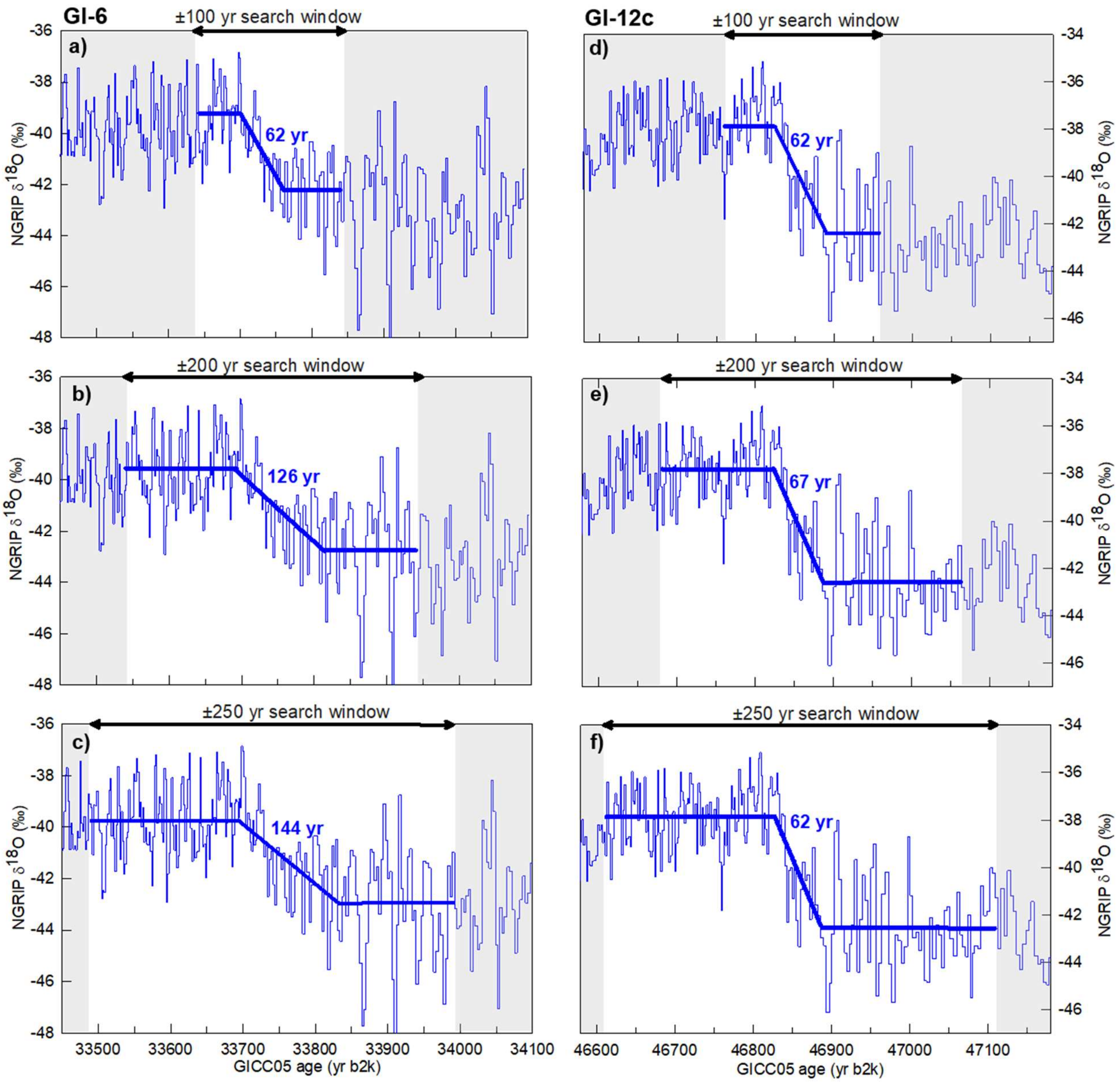

Supplementary Figure 4. Scatter plots of the NEEM transition duration vs NGRIP transition duration for (a) $\delta^{18}O$ (blue squares), (b) d-excess (red triangles), (c) $[Ca^{2+}]$ (green dots), (d) $[Na^{+}]$ (purple diamonds). In e) all tracers ($\delta^{18}O$ in plain blue triangle, d-excess in opened blue triangle, $[Ca^{2+}]$ in plain pink circle and $[Na^{+}]$ in open pink circles). Marginal posterior 5-95% credible intervals are also indicated (light grey). A 1:1 line is added in dark grey in each graph.

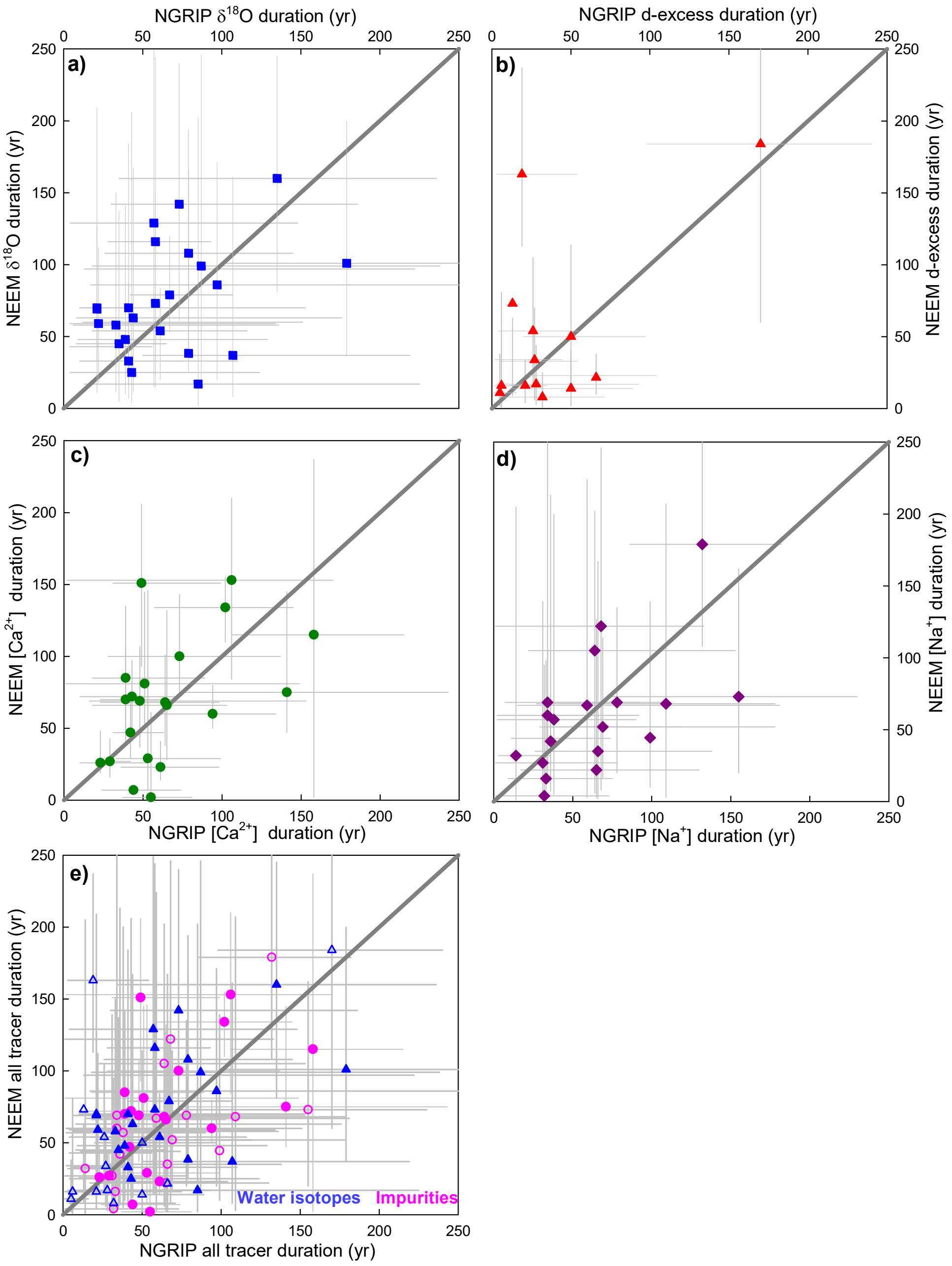

Supplementary Figure 5. Duration estimates of the transitions in NEEM $\delta^{18}O$ (blue) and δD (grey) into each studied GI.

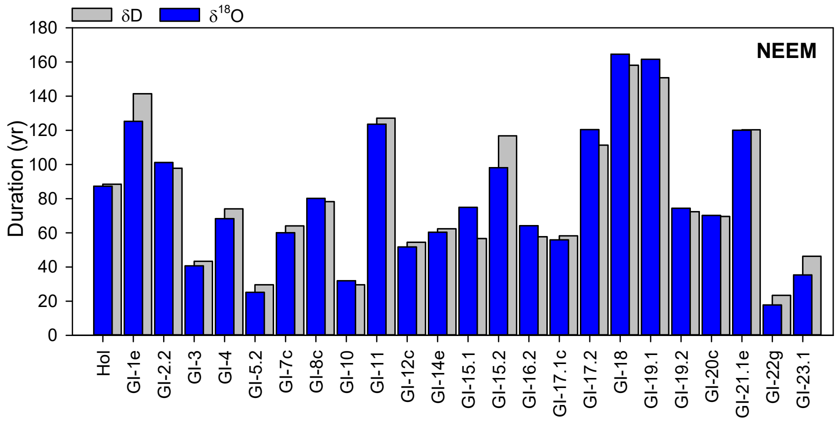

Supplementary Figure 6. Comparison of the orbital-scale climatic background and the transition durations in NGRIP and NEEM ice-core tracers. Marine Isotope Stages (MIS) are indicated by the grey bars; MIS boundaries are defined following Lisiecki and Raymo (2005). (a) 65°N summer insolation (grey, ref. 4) and atmospheric CO2 concentration composite record from Antarctic ice cores (green, ref. 5); (b) Benthic foraminifera $\delta^{18}O$ composite (light blue, ref. 6) and Red Sea Relative Sea Level (RSL) (probability maximum, dark blue, ref. 7); (c) NGRIP transition durations in $\delta^{18}O$ (blue), d-excess (red), $[Ca^{2+}]$ (green), $[Na^{+}]$ (purple) and annual layer thickness (λ, black) superimposed onto the NGRIP $\delta^{18}O$ record (grey, ref. 8). (d) NEEM transition durations in $\delta^{18}O$ (light blue), d-excess (orange), $[Ca^{2+}]$ (khaki green) and $[Na^{+}]$ (pink) also superimposed onto the NGRIP $\delta^{18}O$ record (grey).

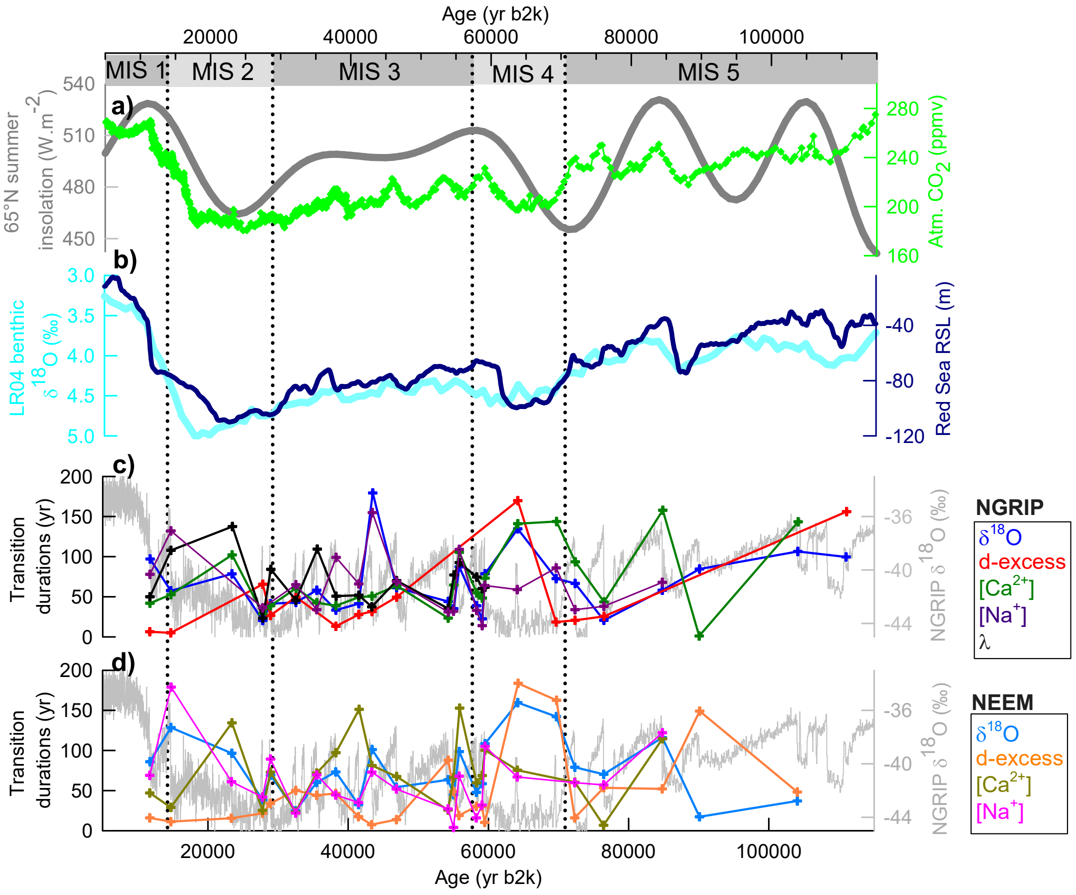

Supplementary Figure 7. Simulated climatic time series with unforced abrupt oscillations: surface air temperature (blue) and precipitation rate (grey) both at the model grid point closest to NGRIP, sea-ice extent in the Irminger Seas (brown) and NAO index (pink). Onsets, end points (symbols) and ramps (oblique lines) together with associated uncertainty intervals (horizontal shaded lines) found by the ramp-fitting analyses (this study) are indicated. Note that the time axis is reversed in order to ease the comparison with ice-core data time series.

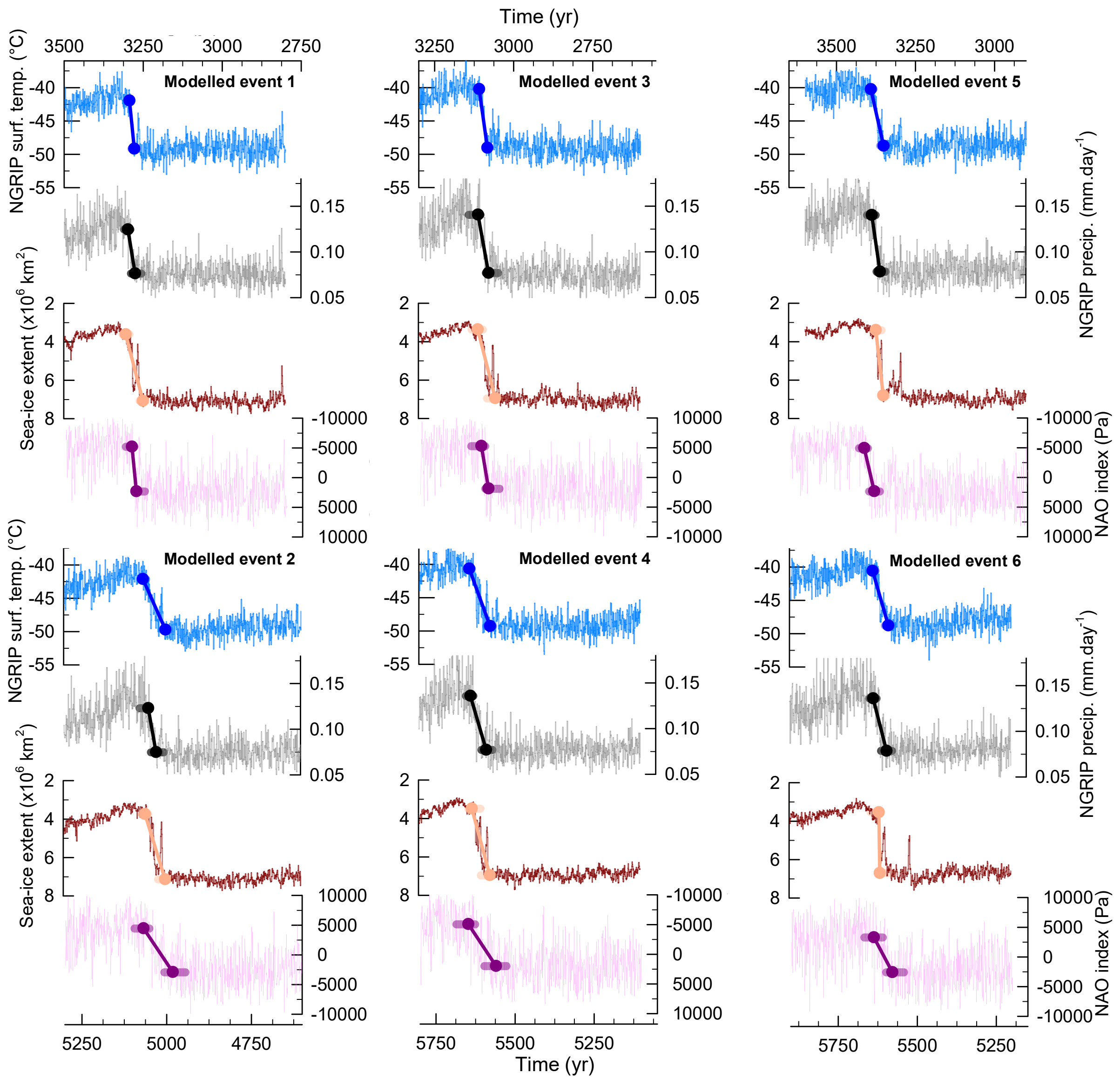

Supplementary Figure 8. Simulated stadial (a) and interstadial (b) sea-ice concentration under prescribed atmospheric CO_2 concentrations of 185 ppm in the low-resolution version of CCSM4.

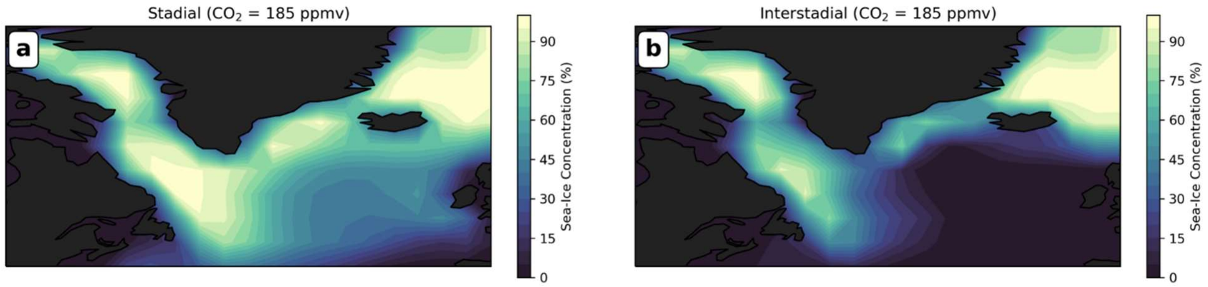

Supplementary Figure 9. Empirical Orthogonal Function (EOF) analysis on the simulated North Atlantic Oscillation (NAO) under prescribed atmospheric CO_2 concentrations of 185 ppm in the low-resolution version of CCSM4, with (a) EOF1, (b) EOF2 and (c) EOF3 and the associated explained variance (var). (d-f) Time series of the Principal Components 1, 2 and 3 across the unforced abrupt event 1.

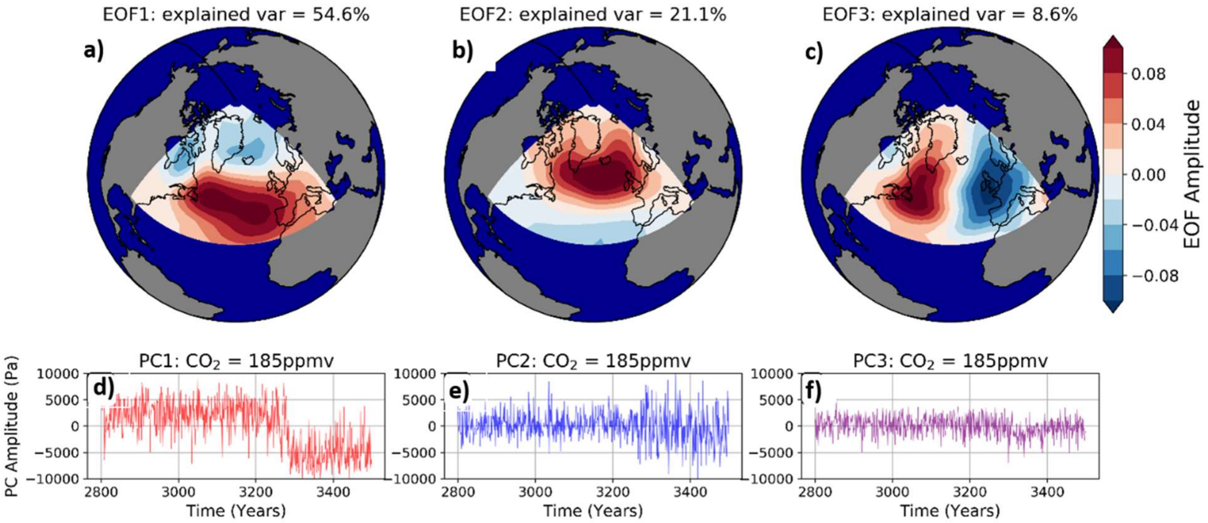

Supplementary references

1 Gkinis, V., Simonsen, S. B., Buchardt, S. L., White, J. W. C. & Vinther, B. M. Water isotope diffusion rates from the NorthGRIP ice core for the last 16,000 years – Glaciological and paleoclimatic implications. *Earth and Planetary Science Letters* **405**, 132-141. https://doi.org/110.1016/j.epsl.2014.1008.1022 (2014).

2 Erhardt, T. *et al.* Decadal-scale progression of the onset of Dansgaard–Oeschger warming events. *Climate of the Past* **15**, 811–825 (2019).

3 Svensson, A. *et al.* A 60 000 year Greenland stratigraphic ice core chronology. *Clim. Past* **4**, 47-57, doi:10.5194/cp-4-47-2008 (2008).

4 Laskar, J. *et al.* A long-term numerical solution for the insolation quantities of the Earth. *A&A* **428**, 261–285 (2004).

5 Bereiter, B. *et al.* Revision of the EPICA Dome C CO2 record from 800 to 600 kyr before present. *Geophysical Research Letters* **42**, 542-549, doi:10.1002/2014GL061957 (2015).

6 Lisiecki, L. E. & Raymo, M. E. Plio-Pleistocene Stack of 57 Globally Distributed Benthic d^{18}O Records. *Paleoceanography* **20**, doi:10.1029/2004PA001071 (2005).

7 Grant, K. M. *et al.* Sea-level variability over five glacial cycles. *Nature Communications* **5**, 5076, doi:10.1038/ncomms6076 (2014).

8 NorthGRIP-community-members. High-resolution record of Northern Hemisphere climate extending into the last interglacial period. *Nature* **431**, 147-151 (2004).
