# Supplementary material for: The anatomy of past abrupt warmings recorded in Greenland ice
Source: Nat Commun. 2021 Apr 8;12:2106. doi: 10.1038/s41467-021-22241-w (PMC8032679; doi:10.1038/s41467-021-22241-w)
Supplement: Supplementary file 3 — Description of Additional Supplementary Files [file 41467_2021_22241_MOESM3_ESM.pdf]

Description of Additional Supplementary Files

File Name: Supplementary Data 1
Description: *NGRIP high-resolution d-excess data*

File Name: Supplementary Data 2
Description: *NGRIP & NEEM high-resolution log (Ca^{2+}) and log (Na^{+}) data*

File Name: Supplementary Data 3
Description: *NGRIP annual layer thickness data*

File Name: Supplementary Data 4
Description: Ages (yr b2k) of the onset, t2, and end, t1, and equivalent depths (m), d2 and d1 respectively, of the studied transitions together with their durations and associated uncertainty intervals (marginal posterior 5-95% credible intervals) found by the ramp-fitting analysis on NGRIP and NEEM ice-core tracers.

File Name: Supplementary Code 1
Description: *Folder including all files related to, and necessary to run the rampfitting model*
