# Supplementary material for: The anatomy of past abrupt warmings recorded in Greenland ice
Source: Nat Commun. 2021 Apr 8;12:2106. doi: 10.1038/s41467-021-22241-w (PMC8032679; doi:10.1038/s41467-021-22241-w)
Supplement: Supplementary file 8 — Supplementary Code 1 [file 41467_2021_22241_MOESM8_ESM.zip › SupplementaryCode1/html documentation and examples/ex_linefit.html]

Fitting a line 

# Fitting a line

This demo follows the linefit example of EMCEE for python. See full description here: http://dan.iel.fm/emcee/current/user/line/

## Contents

- Generate synthetic data
- Least squares fit
- Likelihood
- Prior information
- Find the posterior distribution using GWMCMC
- Apply the hammer:
- Auto-correlation function
- Corner plot of parameters
- Plot of posterior fit

## Generate synthetic data

First we generate some noisy data which falls on a line. We know the true parameters of the line and the parameters of the noise added to the observations.

In this surrogate data there are two sources of uncertainty. One source with known variance (yerr), and another multiplicative uncertainty with unknown variance.

```
% This is the true model parameters used to generate the noise
m_true = [-0.9594;4.294;log(0.534)]

N = 50;
x = sort(10*rand(1,N));
yerr = 0.1+0.5*rand(1,N);
y = m_true(1)*x+m_true(2);
y = y + abs(exp(m_true(3))*y) .* randn(1,N);
y = y + yerr .* randn(1,N);


close all %close all figures
errorbar(x,y,yerr,'ks','markerfacecolor',[1 1 1]*.4,'markersize',4);
axis tight
```

```
m_true =
      -0.9594
        4.294
     -0.62736
```

## Least squares fit

lscov can be used to fit a straight line to the data assuming that the errors in yerr are correct. Notice how this results in very optimistic uncertainties on the slope and intercept. This is because this method only accounts for the known source of error.

```
[m_lsq,sigma_mlsq,MSE]=lscov([x;ones(size(x))]',y',diag(yerr.^2));
sigma_m_lsq=sigma_mlsq/sqrt(MSE); %see help on lscov
m_lsq
sigma_m_lsq

hold on
plot(x,polyval(m_lsq,x),'b--','linewidth',2)
legend('Data','LSQ fit')
```

```
m_lsq =
      -1.0692
       4.4279
sigma_m_lsq =
     0.011028
     0.076933
```

## Likelihood

We define a likelihood function consistent with how the data was generated, and then we use fminsearch to find the max-likelihood fit of the model to the data.

```
% First we define a helper function equivalent to calling log(normpdf(x,mu,sigma))
% but has higher precision because it avoids truncation errors associated with calling
% log(exp(xxx)).
lognormpdf=@(x,mu,sigma)-0.5*((x-mu)./sigma).^2  -log(sqrt(2*pi).*sigma);

forwardmodel=@(m)m(1)*x + m(2);
variancemodel=@(m) yerr.^2 + (forwardmodel(m)*exp(m(3))).^2;

logLike=@(m)sum(lognormpdf(y,forwardmodel(m),sqrt(variancemodel(m))));

m_maxlike=fminsearch(@(m)-logLike(m),[polyfit(x,y,1) 0]');
```

## Prior information

Here we formulate our prior knowledge about the model parameters. Here we use flat priors within a hard limits for each of the 3 model parameters. GWMCMC allows you to specify these kinds of priors as logical expressions.

```
logprior =@(m) (m(1)>-5)&&(m(1)<0.5) && (m(2)>0)&&(m(2)<10) && (m(3)>-10)&&(m(3)<1) ;
```

## Find the posterior distribution using GWMCMC

Now we apply the MCMC hammer to draw samples from the posterior.

```
% first we initialize the ensemble of walkers in a small gaussian ball
% around the max-likelihood estimate.
minit=bsxfun(@plus,m_maxlike,randn(3,100)*0.01);
```

## Apply the hammer:

Draw samples from the posterior

```
tic
m=gwmcmc(minit,{logprior logLike},100000,'ThinChain',5,'burnin',.2);
toc
```

```
Elapsed time is 6.605606 seconds.
```

## Auto-correlation function

```
figure
[C,lags,ESS]=eacorr(m);
plot(lags,C,'.-',lags([1 end]),[0 0],'k');
grid on
xlabel('lags')
ylabel('autocorrelation');
text(lags(end),0,sprintf('Effective Sample Size (ESS): %.0f_ ',ceil(mean(ESS))),'verticalalignment','bottom','horizontalalignment','right')
title('Markov Chain Auto Correlation')
```

## Corner plot of parameters

```
figure
ecornerplot(m,'ks',true,'color',[.6 .35 .3])
```

## Plot of posterior fit

```
figure
m=m(:,:)'; %flatten the chain

%plot 100 samples...
for kk=1:100
    r=ceil(rand*size(m,1));
    h=plot(x,forwardmodel(m(r,:)),'color',[.6 .35 .3].^.3);
    hold on
end
h(2)=errorbar(x,y,yerr,'ks','markerfacecolor',[1 1 1]*.4,'markersize',4);

h(4)=plot(x,forwardmodel(m_lsq),'b--','linewidth',2);
h(3)=plot(x,forwardmodel(median(m)),'color',[.6 .35 .3],'linewidth',3);
h(5)=plot(x,forwardmodel(m_true),'r','linewidth',2);

axis tight
legend(h,'Samples from posterior','Data','GWMCMC median','LSQ fit','Truth')
```

Published with MATLAB® R2015a
