# Supplementary material for: The anatomy of past abrupt warmings recorded in Greenland ice
Source: Nat Commun. 2021 Apr 8;12:2106. doi: 10.1038/s41467-021-22241-w (PMC8032679; doi:10.1038/s41467-021-22241-w)
Supplement: Supplementary file 8 — Supplementary Code 1 [file 41467_2021_22241_MOESM8_ESM.zip › SupplementaryCode1/html documentation and examples/ex_behappy.html]

Don't worry, be Happy 

# Don't worry, be Happy

Sampling a smiley face likelihood function.

## Contents

- Smiley face equation
- Draw samples from the distribution using GWMCMC
- Important links

## Smiley face equation

Formulate a likelihood function inspired by an equation of a smiley face.

Source Michael Borcherds: https://twitter.com/mike\_geogebra/status/135391208703930369

```
logHappiness=@(m)1-exp(1e-4*((m(1)^4+2*m(1)^2*m(2)^2-0.3*m(1)^2*m(2)-40.75*m(1)^2+m(2)^4-m(2)^3-40.75*m(2)^2+25*m(2)+393.75)*((m(1)+3)^2+(m(2)-7)^2-1)*((m(1)-3)^2+(m(2)-7)^2-1)*(m(1)^2+(m(2)-2)^2-64)));
```

## Draw samples from the distribution using GWMCMC

Now we apply the MCMC hammer to draw samples from the logHappiness distribution.

```
[models,logP]=gwmcmc(randn(2,100),logHappiness,100000,'ThinChain',2);
models(:,:,1:end*.2)=[];
models=models(:,:)';


plot(models(:,1),models(:,2),'yo','markerfacecolor',[1 1 0]*.8);

axis equal off


title('GWMCMC says: "Don''t Worry, Be Happy!"');
```

## Important links

Bobby McFerrin on youtube: https://www.youtube.com/watch?v=d-diB65scQU

Published with MATLAB® R2015a
