# Supplementary material for: The anatomy of past abrupt warmings recorded in Greenland ice
Source: Nat Commun. 2021 Apr 8;12:2106. doi: 10.1038/s41467-021-22241-w (PMC8032679; doi:10.1038/s41467-021-22241-w)
Supplement: Supplementary file 8 — Supplementary Code 1 [file 41467_2021_22241_MOESM8_ESM.zip › SupplementaryCode1/html documentation and examples/ex_breakfit.html]

Fitting a trend-change model to a time series 

# Fitting a trend-change model to a time series

This code fits a trend-change model to a historical time series of sea level in Amsterdam with gaps.

## Contents

- Input data
- Define trend change forward model:
- Make an initial guess for the model parameters.
- Likelihood
- Prior information
- Find the posterior distribution using GWMCMC
- Apply the hammer:
- Plot the auto-correlation function
- Corner plot of parameters
- Plot of posterior fit

## Input data

Amsterdam sea level from this source: http://www.psmsl.org/data/longrecords/

```
Y=[1700 -152; 1701 -158; 1702 -132; 1703 -172; 1704 -135; 1705 -167; 1706 -192; 1707 -153; 1708 -149; 1709 -187; 1710 -168; 1711 -140; 1712 -129; 1713 -151;
   1714 -106; 1715 -172; 1716 -168; 1717 -164; 1718 -185; 1719 -182; 1720 -109; 1721 -146; 1722 -141; 1723 -99; 1724 -145; 1725 -166; 1726 -108; 1727 -136;
   1728 -195; 1729 -176; 1730 -148; 1731 -108; 1732 -134; 1733 -160; 1734 -165; 1735 -181; 1736 -109; 1737 -92; 1738 -152; 1739 -123; 1740 -124; 1741 -122;
   1742 -154; 1743 -144; 1744 -148; 1745 -178; 1746 -178; 1747 -142; 1748 -147; 1749 -167; 1766 -175; 1767 -111; 1768 -160; 1769 -86; 1770 -94; 1771 -87;
   1772 -142; 1773 -143; 1774 -135; 1775 -127; 1776 -150; 1777 -131; 1778 -155; 1779 -131; 1780 -130; 1781 -134; 1782 -160; 1783 -157; 1784 -173; 1785 -178;
   1786 -178; 1787 -125; 1788 -204; 1789 -161; 1790 -109; 1791 -92; 1792 -150; 1793 -154; 1794 -118; 1795 -121; 1796 -157; 1797 -134; 1798 -135; 1799 -177;
   1800 -175; 1801 -90; 1802 -159; 1803 -172; 1804 -130; 1805 -142; 1806 -106; 1807 -105; 1808 -183; 1809 -151; 1810 -128; 1811 -137; 1812 -141; 1813 -150;
   1814 -185; 1815 -144; 1816 -113; 1817 -102; 1818 -160; 1819 -158; 1820 -194; 1821 -123; 1822 -125; 1823 -198; 1824 -97; 1825 -87; 1826 -126; 1827 -97;
   1828 -124; 1829 -119; 1830 -141; 1831 -94; 1832 -141; 1833 -106; 1834 -77; 1835 -105; 1836 -96; 1837 -88; 1838 -117; 1839 -114; 1840 -111; 1841 -85;
   1842 -132; 1843 -57; 1844 -53; 1845 -90; 1846 -80; 1847 -118; 1848 -141; 1849 -101; 1850 -91; 1851 -102; 1852 -97; 1853 -113; 1854 -49; 1855 -111;
   1856 -85;  1857 -145; 1858 -137; 1859 -102; 1860 -113; 1861 -94; 1862 -125; 1863 -121; 1864 -161; 1865 -157; 1866 -93; 1867 -58; 1868 -91; 1869 -75; 1870 -129;
   1871 -141; 1874 -110; 1875 -125; 1876 -80; 1877 -43; 1878 -60; 1879 -79; 1880 -31; 1881 -64; 1882 -74; 1883 -58; 1884 -54; 1885 -75; 1886 -88; 1887 -64; 1888 -86;
   1889 -53; 1890 -84; 1891 -94; 1892 -78; 1893 -67; 1894 -92; 1895 -74; 1896 -81; 1897 -82; 1898 -32; 1899 -36; 1900 -67; 1901 -45; 1902 -62; 1903 -25; 1904 -58; 1905 -32;
   1906 -34; 1907 -75; 1908 -66; 1909 -36; 1910 -12; 1911 -24; 1912 -7; 1913 -22; 1914 0; 1915 7; 1916 -5; 1917 -37; 1918 -44; 1919 -38; 1920 14; 1921 -10;
   1922 -16; 1923 -38;1925 29];
t=Y(:,1);
Y=Y(:,2);
```

## Define trend change forward model:

```
forwardmodel=@(t,m)(m(1)*(t<m(3))+m(2)*(t>m(3))).*(t-m(3))+m(4);
```

## Make an initial guess for the model parameters.

```
p=polyfit(t-mean(t),Y,1);
m0=[p(1) p(1) mean(t) p(2)]';
sigma=std(Y-forwardmodel(t,m0));
m0=[m0 ; log(sigma)];
```

## Likelihood

We assume the data are normally distributed around the forward model.

```
% First we define a helper function equivalent to calling log(normpdf(x,mu,sigma))
% but has higher precision because it avoids truncation errors associated with calling
% log(exp(xxx)).
lognormpdf=@(x,mu,sigma)-0.5*((x-mu)./sigma).^2  -log(sqrt(2*pi).*sigma);


logLike=@(m)sum(lognormpdf(y,forwardmodel(t,m),m(5)));
```

## Prior information

We want to restrict the model to place the kink-point within the observed time interval. All other parameters have a uniform prior.

```
logprior = @(m)(m(3)>min(t))&(m(3)<max(t));
```

## Find the posterior distribution using GWMCMC

Now we apply the MCMC hammer to draw samples from the posterior.

```
% first we initialize the ensemble of walkers in a small gaussian ball
% around the m0 estimate.

ball=randn(length(m0),30)*0.1;
ball(:,3)=ball(:,3)*200;
mball=bsxfun(@plus,m0,ball);
```

## Apply the hammer:

Draw samples from the posterior.

```
tic
m=gwmcmc(mball,{logprior logL},300000,'burnin',.3,'stepsize',2);
toc
```

```
Elapsed time is 25.385783 seconds.
```

## Plot the auto-correlation function

And determine the effective sample size.

```
figure
[C,lags,ESS]=eacorr(m);
plot(lags,C,'.-',lags([1 end]),[0 0],'k');
grid on
xlabel('lags')
ylabel('autocorrelation');
text(lags(end),0,sprintf('Effective Sample Size (ESS): %.0f_ ',ceil(mean(ESS))),'verticalalignment','bottom','horizontalalignment','right')
title('Markov Chain Auto Correlation')
```

## Corner plot of parameters

The corner plot shows a bi-modal distribution with two different places you might place the kink in the trend-change model.

```
figure
ecornerplot(m,'ks',true,'color',[.6 .35 .3],'names',{'rate_1' 'rate_2' 'kink' 'k' '\sigma'})
```

## Plot of posterior fit

```
figure
m=m(:,:)'; %flatten the chain


%make a 2d histogram of forwardmodel of the posterior samples
ygrid=linspace(min(Y),max(Y),200);
tgrid=min(t):max(t);
Ycount=zeros(length(ygrid),length(tgrid));
for kk=1:1000
    r=ceil(rand*size(m,1));
    Ymodel=forwardmodel(tgrid,m(r,:));
    Ybin=round((Ymodel-ygrid(1))*length(ygrid)/(ygrid(end)-ygrid(1)));
    for jj=1:length(tgrid)
        Ycount(Ybin(jj),jj)	=Ycount(Ybin(jj),jj)+1;
    end
end
Ycount(Ycount==0)=nan;
h=imagesc(Ycount,'Xdata',tgrid,'Ydata',ygrid);
axis xy

hold on

h=plot(t,Y,'ks','markersize',5);

[~, mm]=kmeans(m, 2); %use Kmeans to characterize two solutions

h(2)=plot(tgrid,forwardmodel(tgrid,mm(1,:)),'color',[.6 .45 .3],'linewidth',2);
h(3)=plot(tgrid,forwardmodel(tgrid,mm(2,:)),'color',[.6 .3 .45],'linewidth',2);

axis tight
legend(h,'Data','Model A','Model B','location','best')
```

Published with MATLAB® R2015a
