# Supplementary material for: The anatomy of past abrupt warmings recorded in Greenland ice
Source: Nat Commun. 2021 Apr 8;12:2106. doi: 10.1038/s41467-021-22241-w (PMC8032679; doi:10.1038/s41467-021-22241-w)
Supplement: Supplementary file 8 — Supplementary Code 1 [file 41467_2021_22241_MOESM8_ESM.zip › SupplementaryCode1/html documentation and examples/ex_rosenbrockbanana.html]

The MCMC hammer 

# The MCMC hammer

GWMCMC is an implementation of the Goodman and Weare 2010 Affine invariant ensemble Markov Chain Monte Carlo (MCMC) sampler. MCMC sampling enables bayesian inference. The problem with many traditional MCMC samplers is that they can have slow convergence for badly scaled problems, and that it is difficult to optimize the random walk for high-dimensional problems. This is where the GW-algorithm really excels as it is affine invariant. It can achieve much better convergence on badly scaled problems. It is much simpler to get to work straight out of the box, and for that reason it truly deserves to be called the MCMC hammer.

See also: http://astrobites.org/2012/02/20/code-you-can-use-the-mcmc-hammer/

## Contents

- Rosenbrock: A badly scaled example
- Apply the MCMC hammer:
- References:

## Rosenbrock: A badly scaled example

A classical difficult low dimensional problem is the rosenbrock density. It is defined by the following log-probability function:

```
logPfun=@(m) -(100*(m(2,:)-m(1,:).^2).^2 +(1-m(1,:)).^2)/20;

%lets visualize it:
close all
[X,Y]=meshgrid(-4:.01:6,-1:.02:34);
Z=logPfun([X(:) Y(:)]'); Z=reshape(Z,size(X));
contour(X,Y,exp(Z))
colormap(parula)
title('The Rosenbrock banana')
xlim([-4 6])
ylim([-1 34])
```

## Apply the MCMC hammer:

Now we apply the Goodman & Weare MCMC sampler and plot the results on top

```
M=2; %number of model parameters
Nwalkers=40; %number of walkers/chains.
minit=randn(M,Nwalkers);
tic
models=gwmcmc(minit, logPfun,100000,'StepSize',30,'burnin',.2);
toc


%flatten the chain: analyze all the chains as one

models=models(:,:);

%plot the results

hold on
plot(models(1,:),models(2,:),'k.')

legend('Rosenbrock','GWMCMC samples','location','northwest')
```

```
Elapsed time is 2.228519 seconds.
```

## References:

- Goodman & Weare (2010), Ensemble Samplers With Affine Invariance, Comm. App. Math. Comp. Sci., Vol. 5, No. 1, 65ï¿½80
- Foreman-Mackey, Hogg, Lang, Goodman (2013), emcee: The MCMC Hammer, arXiv:1202.3665

-Aslak Grinsted 2015

Published with MATLAB® R2015a
