# Supplementary figures and images for: The anatomy of past abrupt warmings recorded in Greenland ice
Source: Nat Commun. 2021 Apr 8;12:2106. doi: 10.1038/s41467-021-22241-w (PMC8032679; doi:10.1038/s41467-021-22241-w)

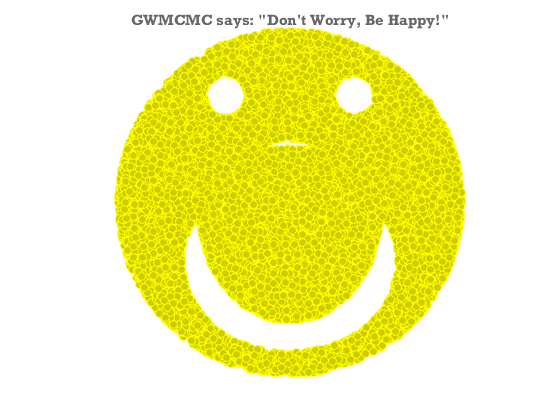

Supplement: Supplementary file 8 — Supplementary Code 1 [file 41467_2021_22241_MOESM8_ESM.zip › SupplementaryCode1/html documentation and examples/ex_behappy_01.png]

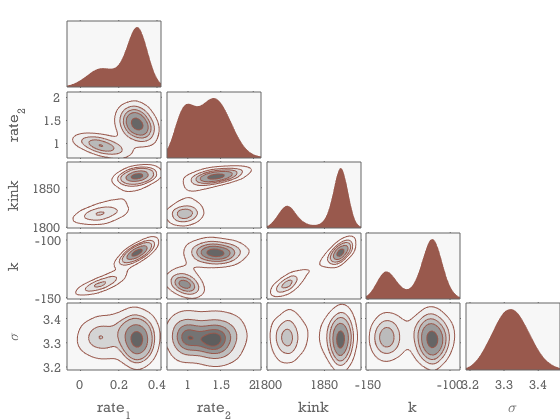

Supplement: Supplementary file 8 — Supplementary Code 1 [file 41467_2021_22241_MOESM8_ESM.zip › SupplementaryCode1/html documentation and examples/ex_breakfit_02.png]

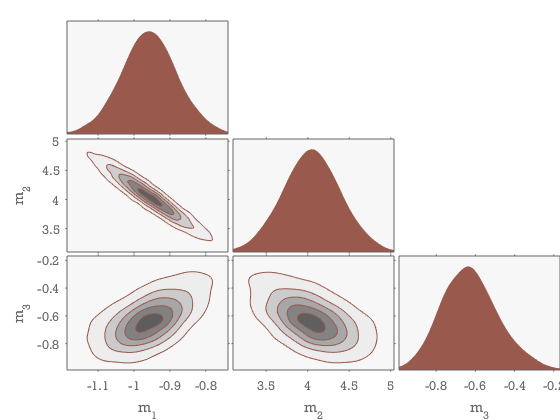

Supplement: Supplementary file 8 — Supplementary Code 1 [file 41467_2021_22241_MOESM8_ESM.zip › SupplementaryCode1/html documentation and examples/ex_linefit_04.png]

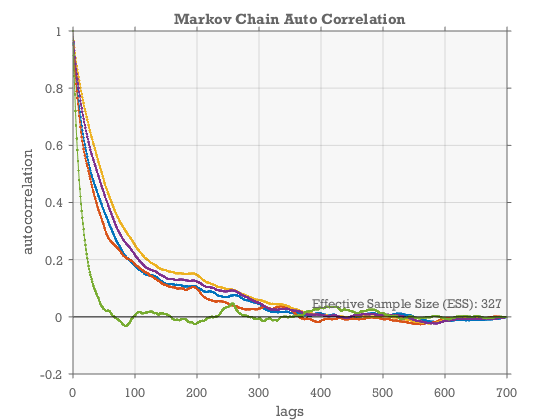

Supplement: Supplementary file 8 — Supplementary Code 1 [file 41467_2021_22241_MOESM8_ESM.zip › SupplementaryCode1/html documentation and examples/ex_breakfit_01.png]

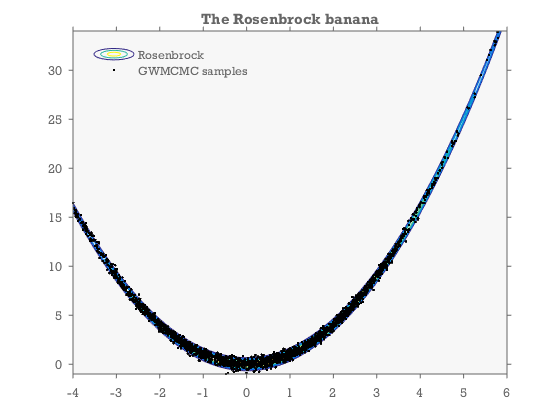

Supplement: Supplementary file 8 — Supplementary Code 1 [file 41467_2021_22241_MOESM8_ESM.zip › SupplementaryCode1/html documentation and examples/ex_rosenbrockbanana_02.png]

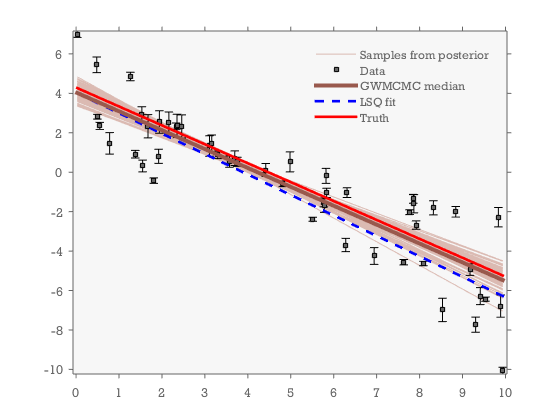

Supplement: Supplementary file 8 — Supplementary Code 1 [file 41467_2021_22241_MOESM8_ESM.zip › SupplementaryCode1/html documentation and examples/ex_linefit_05.png]

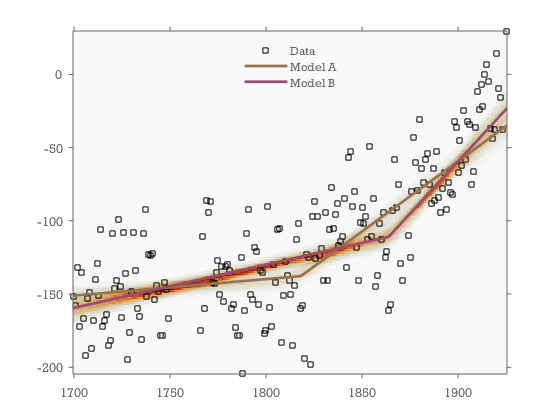

Supplement: Supplementary file 8 — Supplementary Code 1 [file 41467_2021_22241_MOESM8_ESM.zip › SupplementaryCode1/html documentation and examples/ex_breakfit_03.png]

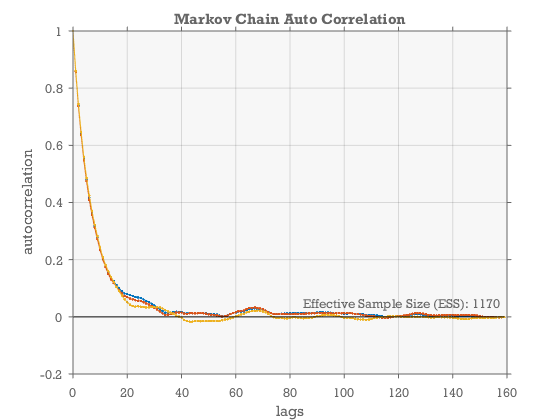

Supplement: Supplementary file 8 — Supplementary Code 1 [file 41467_2021_22241_MOESM8_ESM.zip › SupplementaryCode1/html documentation and examples/ex_linefit_03.png]

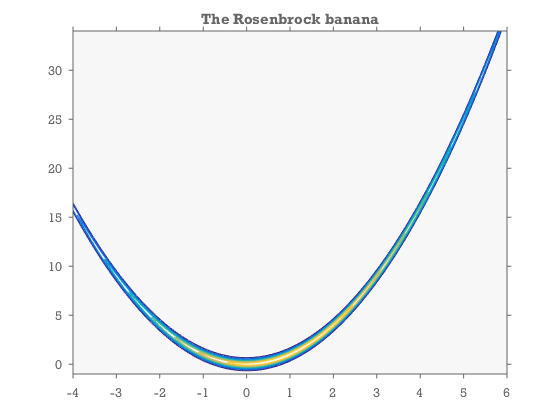

Supplement: Supplementary file 8 — Supplementary Code 1 [file 41467_2021_22241_MOESM8_ESM.zip › SupplementaryCode1/html documentation and examples/ex_rosenbrockbanana_01.png]

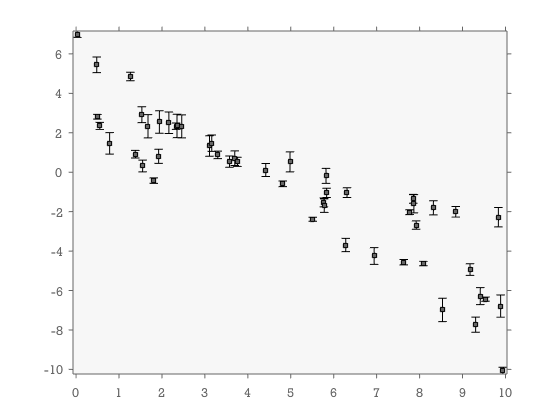

Supplement: Supplementary file 8 — Supplementary Code 1 [file 41467_2021_22241_MOESM8_ESM.zip › SupplementaryCode1/html documentation and examples/ex_linefit_01.png]

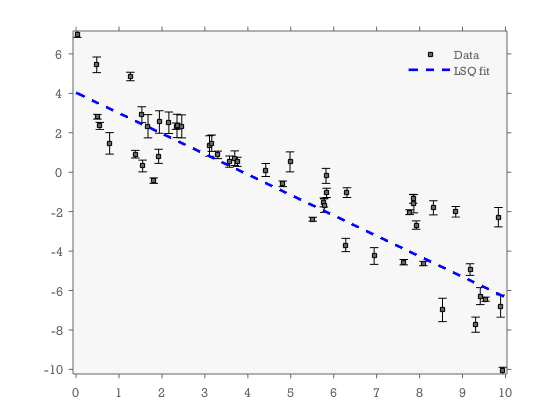

Supplement: Supplementary file 8 — Supplementary Code 1 [file 41467_2021_22241_MOESM8_ESM.zip › SupplementaryCode1/html documentation and examples/ex_linefit_02.png]
